# The Optimization of Renal Graft Preservation Temperature to Mitigate Cold Ischemia-Reperfusion Injury in Kidney Transplantation

**DOI:** 10.3390/ijms24010567

**Published:** 2022-12-29

**Authors:** Maria Abou Taka, George J. Dugbartey, Alp Sener

**Affiliations:** 1 Department of Microbiology and Immunology, Schulich School of Medicine and Dentistry, Western University, London, ON N6A 5C1, Canada; 2 Matthew Mailing Centre for Translational Transplant Studies, London Health Sciences Centre, London, ON N6A 5A5, Canada; 3 Department of Surgery, Division of Urology, London Health Sciences Centre, London, ON N6A 5A5, Canada; 4 Multi-Organ Transplant Program, London Health Sciences Centre, London, ON N6A 5A5, Canada; 5 Department of Pharmacology and Toxicology, School of Pharmacy, College of Health Sciences, University of Ghana, Legon, Accra P.O. Box LG 1181, Ghana

**Keywords:** renal transplantation, hydrogen sulfide (H_2_S), ischemia-reperfusion injury (IRI), renal graft preservation, static cold storage (SCS)

## Abstract

Renal transplantation is the preferred treatment for patients with end-stage renal disease. The current gold standard of kidney preservation for transplantation is static cold storage (SCS) at 4 °C. However, SCS contributes to renal ischemia-reperfusion injury (IRI), a pathological process that negatively impacts graft survival and function. Recent efforts to mitigate cold renal IRI involve preserving renal grafts at higher or subnormothermic temperatures. These temperatures may be beneficial in reducing the risk of cold renal IRI, while also maintaining active biological processes such as increasing the expression of mitochondrial protective metabolites. In this review, we discuss different preservation temperatures for renal transplantation and pharmacological supplementation of kidney preservation solutions with hydrogen sulfide to determine an optimal preservation temperature to mitigate cold renal IRI and enhance renal graft function and recipient survival.

## 1. Introduction

The global incidence of end-stage renal disease (ESRD) has increased by 35% over the past decade [1]. ESRD is classified as the final stage of chronic kidney disease in which renal function has declined to the point where the kidney is no longer able to support an individual’s daily needs. Patients with ESRD exhibit a glomerular filtration rate of less than 15 mL/min, which is a clinical marker of severe kidney damage [2]. The most common causes of ESRD in North America are diabetes and hypertension, which are chronic diseases often associated with older age. The global prevalence of ESRD and its risk factors is thus predicted to continue rising over the next decade due to the growing senior population [3]. Approximately 5 million people with ESRD require renal replacement therapy in the form of dialysis or kidney transplantation, thus illustrating the significant burden ESRD has on the global healthcare system [4]. Dialysis is particularly effective for removing uremic toxins, achieving electrolyte and pH balance, and resolving body fluid abnormalities [5]. However, it is associated with several long-term risks such as increased infections, cardiovascular complications, and hypotension [6,7]. It has been reported that dialysis carries a 5-year survival rate of 44% along with its significant economic burden [8,9]. In Canada, for example, a 10-year survival rate of patients with ESRD undergoing dialysis is less than 15%, which is comparable to other chronic illnesses such as cancer [10]. Considering the major drawbacks in dialysis, renal transplantation has become the preferred therapeutic option for ESRD patients. Renal transplantation is a surgical procedure that has shown significant growth with recent technological innovations since its inception in the mid-20th century and is now a routine life-saving intervention in clinical practice today. Compared to dialysis, renal transplantation is less costly and is associated with prolonged long-term survival and dramatically enhanced quality of life [11,12]. To illustrate, a 5-year survival rate of renal transplant recipients is approximately 80%, whereas it is only 44% in dialysis patients [11].

## 2. Challenges in Renal Transplantation

### 2.1. Global Donor Kidney Shortage Crisis

Despite it being the established treatment option for patients with ESRD, there are several challenges associated with renal transplantation. One of the greatest challenges in renal transplantation is the global organ shortage crisis due to an ever-increasing number of potential recipients, which ultimately leads to longer kidney transplant waiting lists, increased wait times and the aging ESRD population, all of which lead to high mortality while waiting for life saving organs [12,13]. Without a substantial increase in the number of donor kidneys, relatively fewer patients with ESRD will enjoy the demonstrated benefits of renal transplantation. In Canada, for example, even though just over 1500 kidney transplants were performed in 2020, over 3000 Canadians were still on the transplant waiting list by the end of the year [14]. In that same year, 110 Canadians died while waiting for a kidney transplant, which is a 33% increase from the number of patients who died the year before [14]. The adversities of a growing kidney transplant waiting list is further exacerbated in the United States, where over 105 thousand patients are currently on the national transplant waiting list, with 17 patients dying each day while waiting their turn for a transplant [15]. The growing imbalance between the supply and demand for transplantable kidneys suggests that ESRD patients requiring a kidney transplant face an increasing lifetime probability of never receiving one. This increases mortality rates, as patients with ESRD are forced to undergo longer periods of dialysis while renal transplantation waiting lists continue to rise [15]. The global donor organ shortage crisis has also necessitated the use of suboptimal renal grafts from high-risk donors such as marginal and deceased donors to expand the deceased kidney donor pool to meet the rising demand for renal transplantation. Unfortunately, renal allografts from this source are associated with reduced viability characterized by delayed and/or reduced urine production and filtration capacity, which increases post-transplant complications and mortality [16].

### 2.2. Renal Ischemia-Reperfusion Injury

**Figure 1:**
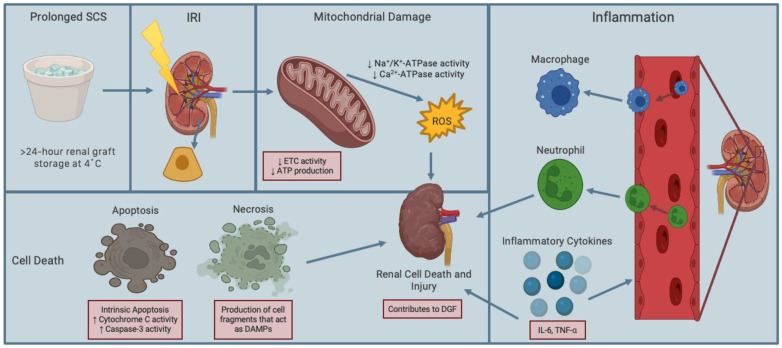
Schematic diagram of renal ischemia-reperfusion injury caused by prolonged static cold storage. Ischemia during renal graft procurement followed by prolonged static cold storage (SCS) results in hypoxia, which decreases the activity of electron transport chain (ETC), leading to electron leakage and decreased production of adenosine triphosphate (ATP). This ultimately increases mitochondrial production of reactive oxygen species (ROS). Reperfusion following ischemia, is characterized by influx of oxygen during restoration of renal blood supply, which further increases ROS production and cell death through necrosis and apoptosis. Thus, ischemia and reperfusion form a paradoxical phenomenon referred to as ischemia-reperfusion injury (IRI). IRI also induces inflammation of the renal tissue through infiltration of neutrophils and macrophages, leading to the release of pro-inflammatory mediators such as cytokines and adhesion molecules. This causes innate immune cells to adhere to the renal endothelium and extravasate to the interstitial space, where they release more damaging ROS, causing further renal cell death and injury. Figure prepared with BioRender (biorender.com).

Another significant challenge in renal transplantation involves renal graft procurement, which is inevitably associated with a pathological process termed renal ischemia-reperfusion injury (IRI). Renal IRI occurs due to temporal cessation of renal blood flow (i.e., ischemia) during the procurement process of the donor kidney and restoration of renal blood supply (i.e., reperfusion) during implantation of the renal graft into the recipient [17]. At the cellular level, hypoxia occurs during ischemia, resulting in a switch from aerobic to anaerobic metabolism, which subsequently decreases production of adenosine triphosphate (ATP), leading to intracellular acidosis associated with increased lactate production (Figure 1). Consequently, lysosomal membranes destabilize, and lysosomal enzymes leak out of the membrane and cause cytoskeletal breakdown [18,19]. This inhibits the activity of Na^+^/K^+^-ATPase (an electrogenic transmembrane protein pump) and Ca^2+^-ATPase pump, causing intracellular accumulation of Na^+^ and water, resulting in cellular edema [19,20]. Moreover, Ca^2+^ excretion decreases, leading to intracellular Ca^2+^ accumulation. In the mitochondria, Ca^2+^ accumulation is associated with overproduction of reactive oxygen species (ROS; a natural by-product of cellular oxidative metabolism that is injurious to cells and tissues) [21]. Furthermore, mitochondria release Ca^2+^ into the cytoplasm, thereby activating Ca^2+^-dependent cytosolic proteases and phospholipids, further contributing to cellular edema. The cellular edema is commonly characterized by formation and opening of mitochondrial permeability transition pores (mPTP; an important indicator of mitochondrial dysfunction and determinant of apoptosis). This occurs along with activation of the intrinsic apoptotic pathway, which includes the translocation of cytochrome c (the initiator protein in apoptosis) and increased activity of caspases-3 (the executioner protein in apoptosis), resulting in apoptotic cell death [22,23].

Following ischemia is reperfusion, when warm oxygenated blood is restored to the ischemic renal graft upon implantation of the donor kidney into the recipient. During reperfusion, there is an influx of molecular oxygen, resulting in further production of ROS [23,24,25,26]. This causes oxidative stress (i.e., the imbalance between ROS generation and antioxidant production) and systemic activation of immune cells (e.g., neutrophils and macrophages) and release of pro-inflammatory mediators (e.g., interleukin-6 and tumor necrosis factor-α, chemokines, adhesion molecules) (Figure 1). This disrupts the cell permeability, resulting in cell death. In the renal vasculature, ROS-induced oxidative stress damages the vascular endothelium through nuclear factor-ĸB activation (NF-ĸB; an inflammation-related transcription factor) and leads to renal cellular apoptosis (Figure 1). Similar to the ischemic phase, intracellular Ca^2+^ levels increase in the mitochondria, which activate calpains (i.e., Ca^2+^-dependent proteases) that damage cellular structures, exacerbating renal tissue injury. Additionally, this leads to the activation of necrosis, which is characterized by cell swelling and subsequent rupture of the cell membrane. This results in the uncontrollable release of cell fragments that act as danger/damage associated molecular patterns (DAMPs) that activate innate and adaptive immune system pathways [27,28]. Together, these pathological processes result in delayed graft function (DGF; the earliest and most frequent post-transplant complications associated with higher rates of acute cellular rejection and shorter renal graft survival) [27,28,29,30]. Overall, the surgical procedure involving renal (and other solid organ) transplantation obligates IRI through loss of energy, disruption of ionic hemostasis, overproduction of ROS, and cell death, which adversely impacts short- and long-term graft survival and increases post-transplant complications.

## 3. Efforts to Mitigate Kidney Transplant-Induced Renal IRI and Improve Renal Graft Quality for Transplantation

### 3.1. Static Cold Storage

Static cold storage (SCS) is currently the gold standard of renal graft preservation. SCS reduces IRI by slowing cellular activity and decreasing the production of toxic metabolites before transplantation, which preserves renal graft quality and reduces long-term post-transplantation complications [31,32]. It involves storage of the renal graft in various preservation solutions, such as University of Wisconsin (UW) solution, on ice at 4 °C prior to kidney transplantation [32]. While short-term SCS of the renal graft reduces cellular metabolism and limits ischemic damage, prolonged periods of SCS (>24 h) hinder the benefits of SCS due to increased microvascular restriction, which increases the rate of IRI and, therefore, DGF and chronic graft failure [33,34]. Hypothermia worsens ischemic injury through reduced ATP production and metabolic activity, as well as reduced Na^+^/K^+^-ATPase activity, which induces osmotic and mitochondrial perturbations. In turn, this decreases renal cell survival, contributing to poorer renal graft function and transplantation outcomes [34]. Therefore, optimization of renal graft preservation temperatures to mitigate cold renal IRI is a critical goal in current kidney transplantation studies.

### 3.2. Hypothermic Machine Perfusion

To limit the negative impact of SCS and expand the pool of kidney donation from high-risk donors, a new preservation technique called hypothermic machine perfusion (HMP) was recently introduced. As with SCS, HMP preserves renal grafts in a preservation solution at 4 °C prior to renal transplantation. However, HMP creates a flow through the kidney that is generated by a pump in a circuit. This circuit allows for preservation solutions to recirculate through the renal vasculature at various temperatures. The continuous perfusion through a pump allows for substantial penetration of preservation solutions within the renal graft, providing efficient oxygen and nutrient delivery to the renal parenchyma and clearance of metabolic waste, while creating a platform for pharmacological interventions [35]. This makes HMP particularly suitable for preservation of renal grafts from marginal and deceased donors. Although HMP has various advantages including lower incidence of DGF compared to SCS, and the ability to predict transplant outcomes based on the parameters it measures (e.g., temperature, pressure, and flow), the equipment it requires is more complicated and expensive than SCS and requires continuous monitoring and evaluation of the renal graft during preservation [35]. Thus, it is no surprise that SCS of renal grafts at 4 °C has been the gold standard of renal preservation.

## 4. Alternatives to Hypothermic Renal Graft Preservation

### 4.1. Normothermic Temperatures at 35–37 °C

Recent studies have focused on potential benefits of normothermic machine perfusion (NMP) of the renal graft at 35–37 °C on graft outcomes and survival. Hosgood et al. [36], for example, examined whether normothermic preservation at 37 °C would enhance function and survival of renal grafts from deceased donors. In their study, declined human DCD (donation-after-circulatory-death) kidneys underwent ex vivo NMP at 37 °C for 60 min. The authors observed improvement in renal graft function following NMP compared those that underwent SCS [37]. Clinically, Minor et al. [37] reported that a human renal graft procured from a marginal donor and subjected to NMP at 35 °C with controlled oxygenated rewarming following 12.5 h of SCS, showed immediate graft function without complications. Considering that NMP is an emerging technology as an important adjunct to renal graft preservation, these findings are of special interest in preservation of renal grafts from marginal or deceased donors, which are known to be highly susceptible to cold ischemic injury. Thus, NMP may provide a protective environment for the graft by ensuring optimal oxygen delivery and supporting metabolic function. However, there are still several limitations of normothermic renal graft preservation. Firstly, NMP, as a modified form of HMP, requires a costly and complex setup that may not be feasible to use in transplantation worldwide. Secondly, it requires renal graft perfusion with red blood cells (RBCs) to ensure sufficient oxygen and nutrient delivery, as kidneys are highly metabolically active at 37 °C. Clinically, third-party packed RBCs in perfusate solutions is complicated, as these RBCs tend to have a limited shelf-life and can be restricted in supply [36,37]. Therefore, future research with clinically approved NMP protocols that addresses the need for blood and the high cost of NMP is essential.

### 4.2. Subnormothermic Temperatures at 20–22 °C

Considering the issues associated with NMP and SCS, perhaps subnormothermic machine perfusion (SMP), a modified form of HMP, may be beneficial for preserving renal grafts with the potential to significantly improve the outcome of marginal or deceased donor kidneys after transplantation. The interest in SMP of renal grafts stems from the idea that cold renal IRI can be prevented at these temperatures without increasing metabolic demand to physiological levels like in NMP and at the same time without inducing SCS-associated hypoxia [38,39,40]. In a porcine model of kidney transplantation, SMP of DCD kidneys at 20 °C for 7 h followed by reperfusion with autologous blood for 2 h preserved structural integrity of the renal graft, and significantly improved renal functional parameters such as renal blood flow, urine production and creatinine clearance compared to SCS and HMP [41]. Previous work by our group also examined renal graft preservation at subnormothermic conditions at 21 °C and 22 °C with Hemopure, a hemoglobin-based oxygen carrier with exceptional oxygen-carrying capacity [42,43]. Following a 4 h reperfusion with autologous blood, we observed upregulation in the expression of pro-survival gene and downregulation of pro-apoptotic and hypoxia-response genes in renal grafts preserved under SMP, which positively correlated with improvement in renal graft outcomes (reduced acute tubular necrosis scores and decreased levels of urinary damage markers and interleukin-6) compared to grafts under SCS [43]. However, like HMP and NMP, the complex renal graft preservation setup at these subnormothermic temperatures still required expensive equipment and significant manpower to ensure active delivery of oxygen and nutrients to the renal grafts.

### 4.3. Subnormothermic Temperatures at 8–10 °C

**Figure 2:**
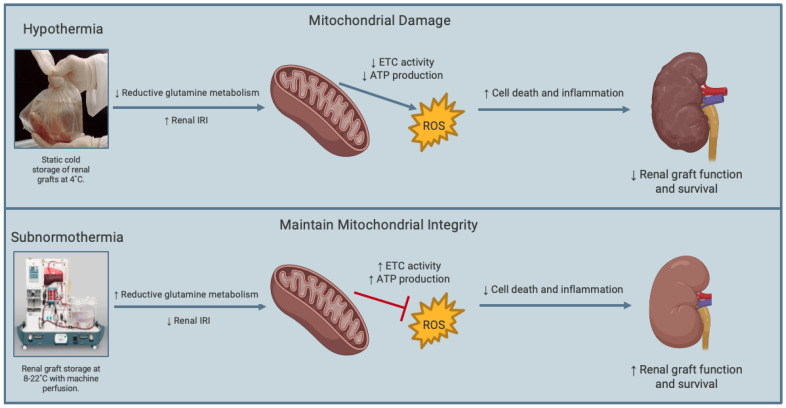
Schematic representation of the effects of subnormothermic and hypothermic renal graft preservation. Compared to static cold storage at 4 °C, subnormothermic temperatures (e.g., 8–22 °C) are associated with increased reductive glutamine metabolism, which reduces renal ischemia-reperfusion injury. In turn, subnormothermia can increase electron transport chain (ETC) activity, which subsequently increases adenosine triphosphate (ATP) production and reduce generation of reactive oxygen species (ROS) to protect the mitochondria and reduce cell death and inflammation. This mechanism enhances renal graft function and survival. Figure prepared with BioRender (biorender.com).

Recent kidney transplantation studies have examined subnormothermic renal graft preservation at 8–10 °C [44,45,46]. These subnormothermic temperatures are of interest because organ metabolism is reduced to the point where oxygen and nutrient demand is not as great as it is to temperatures closer to normal physiologic conditions. Further, it has been shown that crucial cellular and molecular processes that maintain cellular integrity are still active at 8–10 °C, but not at 4 °C, the current gold standard for renal graft preservation (Figure 2). For example, reductive glutamine metabolism is active at ~10 °C, but not at 4 °C, and this process leads to downstream reductive carboxylation [47,48]. This is essential for maintaining redox homeostasis in hypoxic events or mitochondrial defects, as observed in renal IRI. In an experiment to determine the effect of SMP on porcine cardiac grafts, Michel et al. [44] observed that cardiac grafts subjected to 8 °C SMP for 12 h exhibited less ATP depletion, lower endothelin-1 levels and less apoptosis compared to SCS-treated grafts at the same temperature. In the same study, the authors reported greater mitochondrial injury, endothelial cell rupture, and muscle fiber damage in SCS-treated grafts compared to the SMP grafts. Preservation of lung grafts at subnormothermic temperature of 10 °C for 36 h was also found to significantly increase the expression of mitochondrial protective metabolites including itaconate, glutamine and *N*-acetylglutamine compared to lung grafts preserved at the conventional hypothermic temperature of 4 °C [45]. Following analysis of mitochondrial injury markers, the authors further reported that preservation of lung grafts at 10 °C resulted in greater protection of mitochondrial health relative to control grafts preserved at 4 °C [45]. Functional assessments of ex vivo lung perfusion demonstrated that porcine lungs under SMP at 10 °C had higher lung compliances and better oxygenation capabilities, both of which indicate enhanced pulmonary physiology compared to lungs that were stored at the conventional 4 °C storage [45]. The authors clinically applied their preservation technique to human donor lungs and found that preservation of human lung grafts at 10 °C can prolong preservation time to 36 h, while reducing graft dysfunction and post-transplant mechanical ventilation [46]. Although renal graft preservation at 10 °C is yet to be studied, the findings of these studies suggest that preservation of renal grafts at subnormothermic temperatures of 8–10 °C may offer an option to reduce cold renal IRI and DGF, and, thereby, improve graft quality and post-transplant outcomes.

## 5. Supplementing Kidney Preservation Solutions with Pharmacological Additives at Various Temperatures to Mitigate Renal IRI

In addition to determining an optimal preservation temperature to mitigate transplant induced renal IRI, there is a growing interest in pharmacological modifications, such as the addition of amino acids and gasotransmitters, of existing kidney preservation solutions at various temperatures to further prevent renal grafts from IRI. Although conventional preservation solutions contain impermeant agents, osmotic molecules and other important supplements to sustain the low metabolic demand of the graft and prevent cellular edema and ROS production during hypothermic preservation, the increasing incidence of cold IRI and subsequent occurrence of DGF and other post-transplant complications defeat the goal of hypothermic and subnormothermic graft preservation.

### 5.1. Amino Acids and Proteins

**Figure 3:**
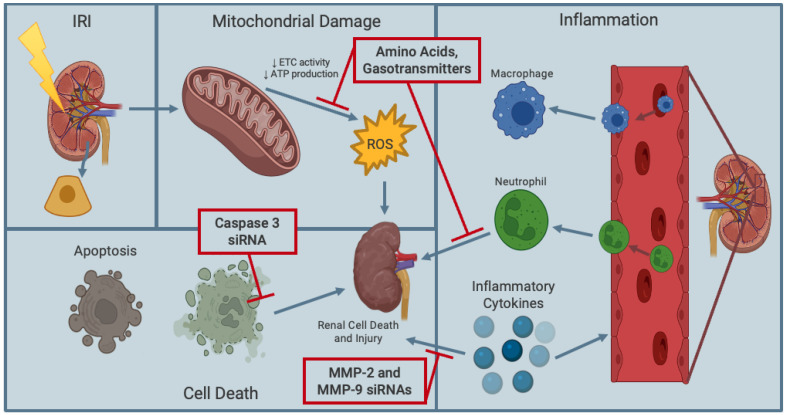
Schematic representation of effective supplementation of kidney preservation solution against renal IRI. Supplementing preservation solutions with amino acids or gasotransmitters can inhibit inflammatory and oxidative stress pathways and prevent the reduction in mitochondrial electron transport chain (ETC) activity and, thus, enhance adenosine triphosphate (ATP) production. Additionally, caspase-3 and MMP-2 and MMP-9 siRNAs can inhibit renal cellular death and inflammatory pathways, respectively, to protect renal grafts against ischemia-reperfusion injury and delayed graft function. Figure prepared with BioRender (biorender.com).

Amino acids are well-established as molecules that can effectively supply nutrients and can act as metabolic substrates in cells. Notably, amino acids can be used as additives in kidney preservation solutions, as they exhibit antioxidant properties that can mitigate renal damage from transplant induced IRI (Figure 3) [49,50]. For instance, in a canine model of renal transplantation, L-arginine, an endogenous substrate for nitric oxide (NO) synthase (an essential cellular signaling molecule part of immune defense mechanisms), was introduced into kidney preservation solution [51]. Histological evaluation of canine kidney structure revealed that L-arginine-supplemented preservation solution resulted in a protective effect on long-term (72-h) hypothermic (4 °C) ischemic damage with reduced lipid peroxidation [51]. Additionally, trophic factors (i.e., proteins that are essential for cell survival) as additives in preservation solutions, such as UW solution, may further protect kidneys from IRI. Kwon et al. [52] investigated trophic factor supplementation of UW solution in an in vitro model of canine kidney transplantation. By supplementing UW solution at 4 °C with trophic factors in primary canine kidney tubule cells, they found a 15% increase in mitochondrial membrane potential, indicative of protected mitochondrial function, and a reduction of caspase-3 enzymatic activity, illustrating a reduction in early apoptosis [52].

In a rat model of renal transplantation, Celic et al. [53] investigated the use of recombinant bone morphogenetic protein-7 (rhBMP-7), which is a member of the transforming growth factor-β superfamily. During embryonic and adult life, the kidney is the major synthesis site of rhBMP-7, which induces the formation of bone at extraskeletal sites [54]. Notably, rhBMP-7 is essential for proper kidney formation and development. Celic et al. [53] perfused rat kidneys with rhBMP-7-supplemented UW solution for 24 h of cold (4 °C) storage and analyzed levels of lipid peroxidation, superoxide dismutase (SOD), and glutathione peroxidase (GSH-Px) using spectrophotometry. Overall, they found increased levels of lipid peroxidation and increased activity of SOD and GSH-Px after 24 h compared to rat kidneys perfused with UW only [53]. Thus, the authors concluded that rhBMP-7-supplemented UW solution at 4 °C protects renal grafts against IRI. Although these findings are promising for mitigating transplant induced IRI, the use of amino acids and proteins as additives in preservation solutions has been limited to hypothermic renal preservation, which is still associated with cold IRI.

### 5.2. Signaling Pathway Inhibitors

Previous studies have demonstrated effective mechanisms of specifically targeting signaling pathways implicated in renal IRI by using small interfering RNAs (siRNAs) [55,56,57,58]. Using a porcine model of kidney transplantation, Yang et al. [55] exogenously introduced naked caspase-3 siRNA into normothermic preservation solutions and found reduced caspase-3 expression levels and, thus, decreased renal cell apoptosis (Figure 3). More recently, Moser et al. investigated the use of matrix metalloproteinases (MMPs) inhibitors that were previously shown to protect human cardiac grafts, which were susceptible to IRI, from damage [56,57,58]. Regarding kidney transplantation, increased levels of MMPs (e.g., MMP-2 and MMP-9) are involved in pathological processes, such as tubular atrophy, fibrosis, basement membrane damage and renal failure [56,57]. Additionally, MMPs are increased in kidney transplant patients with chronic antibody-mediated rejection [57,58]. Moser et al. reported that HMP of rat kidneys with MMP-2 and MMP-9 (i.e., MMPs involved with inflammation associated with acute and chronic renal injury) siRNAs at 4 °C protected the transplanted rat kidneys from IRI. Specifically, MMP-2 and MMP-9 siRNAs inhibited the expression of MMP-2 and MMP-9 and, thus, reduced the incidence of DGF in recipient rat kidneys (Figure 3) [56]. Although these preservation solution additives seem promising for hypothermic kidney storage, future studies should investigate these siRNAs at subnormothermic temperatures, as normothermic kidney preservation is associated with a complex and costly setup, and hypothermic renal graft preservation is correlated with a greater risk of IRI.

### 5.3. Hemopure

Hemoglobin-based oxygen carriers, a type of blood substitute, has also been in the literature as another additive in kidney preservation solutions. This allows for the replacement of autologous blood in renal graft preservation. Recently, Hemopure (Hemoglobin-based oxygen carrier-201, HbO_2_ Therapeutics LLC) has emerged as a promising additive for renal perfusion. Hemopure is bovine-based hemoglobin that exhibits similar oxygen-carrying capacity as human hemoglobin [59]. Aburawi et al. [60] demonstrated that perfusing declined human kidneys for 6 h with Hemopure under normothermic conditions leads to similar renal functional outcomes as perfusing declined human kidneys with packed red blood cells. Studies at our centre have also shown that perfusing porcine kidneys at subnormothermic temperatures of 21 and 22 °C reduced renal graft injury compared to SCS [61]. As such, Hemopure is a promising additive to renal preservation solutions, especially at subnormothermic temperatures.

### 5.4. Gasotransmitters

Recently, gasotransmitters have been applied in kidney preservation solutions at hypothermic and subnormothermic temperatures as a promising therapeutic strategy to mitigate prolonged cold renal IRI (Figure 3). Gasotransmitters are small gaseous signaling molecules that are endogenously produced in mammals. They are present in mammalian tissues at nanomolar to micromolar concentrations and are involved in essential physiological functions such as vasodilation and cellular signaling [62,63]. The first molecule that was classified as a gasotransmitter was NO due to its prominent role as an endothelium-derived relaxing factor [64]. Carbon monoxide (CO) is another gasotransmitter that was subsequently discovered to exhibit cellular signaling and vasodilatory actions like NO [65]. These gasotransmitters are currently under experimental investigation in the context of organ transplantation [66,67,68]. Hosgood et al. [69] previously demonstrated that perfusing porcine kidneys under acute (10 min) normothermic preservation conditions and 16 h SCS with NO and CO provided significant protection against renal IRI by modulating inflammatory and oxidative stress pathways. However, as previously mentioned, normothermic renal graft preservation is complex and costly, and SCS still potentiates the risk of cold renal IRI.

### 5.5. Hydrogen Sulfide as a Gasotransmitter

Another gaseous signaling molecule that is receiving significant experimental attention is hydrogen sulfide (H_2_S). Historically, H_2_S was notoriously known for its toxicity and death at high concentrations through its reversible inhibition of cytochrome c oxidase of the mitochondrial respiratory chain to prevent oxidative phosphorylation and decrease ATP production [70]. However, recent studies have revealed that H_2_S has risen above its negative public image to become an important signaling molecule, possessing therapeutic properties such as antioxidant, anti-inflammatory, anti-apoptotic, anti-thrombotic, vasodilating and pro-angiogenic properties at low physiological concentrations [71]. These essential therapeutic properties of H_2_S suggest that it could be a promising candidate drug in the field of kidney transplantation to mitigate cold renal IRI. Though, H_2_S gas is not ideal to directly administer due to challenges with controlling and obtaining precise concentrations in tissues. As such, H_2_S donors, molecules that release H_2_S, have been of particular interest. Since H_2_S is highly volatile in solutions, ideal H_2_S donors generate H_2_S with different rates of release and duration of treatment [70,71].

Several compounds such as sodium hydrosulfide, GYY4137 and AP39 have been identified as H_2_S donors and experimentally investigated in the context of kidney transplantation [72,73]. Among these, AP39 demonstrated a greater renal graft-protecting potential with improved renal graft function and prolonged recipient survival following its addition to preservation solutions at hypothermic and subnormothermic temperatures [74,75,76,77,78,79]. AP39 is a slow-releasing H_2_S donor molecule that specifically targets the mitochondrion (the major cellular site of transplant-induced IRI) and facilitates H_2_S entry [80]. In addition, our research team demonstrated, in an in vitro model of renal IRI with porcine renal proximal tubular epithelial cells, that exogenous treatment with AP39 significantly reduced ROS production and apoptosis, preserved mitochondrial integrity, and increased cell viability in a dose-dependent manner following 18 h of hypoxia at 21 °C and 24 h of reoxygenation at 37 °C in UW solution [43]. This finding supported our ex vivo observation in the same study with identical preservation conditions using DCD porcine kidneys.

### 5.6. Sodium Thiosulfate—A Clinically Approved H_2_S Donor Drug

Considering that AP39 is still an experimental donor molecule and is, therefore, not clinically viable, our research group began to study a clinically approved H_2_S donor molecule, sodium thiosulfate (STS). STS is currently on the World Health Organization’s list of essential medicines as an antidote for acute cyanide poisoning and as treatments for cisplatin-induced organ toxicity and calciphylaxis in cancer and ESRD patients, respectively [80,81,82,83]. We have recently shown in a rat model of renal transplantation that supplementation of UW solution at 4 °C with STS during SCS elicited protective effects against renal IRI. In comparison with control renal grafts preserved in only UW solution at 4 °C, we observed that renal grafts preserved in STS-supplemented UW solution at 4 °C for 24 h showed preserved renal structures, which were characterized by significantly reduced acute tubular necrosis and apoptosis, as well as downregulation of pro-inflammatory and pro-apoptotic genes [84]. In addition, renal graft function was significantly improved immediately following transplantation, which, ultimately, prolonged recipient survival [84]. However, since there is still a risk of cold renal IRI at 4 °C, future studies should examine the effects of STS-supplemented renal graft preservation at subnormothermic temperatures to reduce renal IRI, DGF, and enhance renal graft function and recipient survival.

## 6. Conclusions

The risk of cold renal IRI has existed with SCS of renal grafts at 4 °C for over half a decade. With the growing incidence of DGF associated with IRI, and the ever-increasing number of patients with ESRD who require renal transplantation, it is critical that we work to shift the paradigm away from SCS at 4 °C to subnormothermic renal graft preservation techniques. Not only would static subnormothermic storage of renal grafts be more cost-effective and feasible for global healthcare systems, but it would also confer a significant advantage of mitochondrial protection and enhance renal graft quality and patient survival. Moreover, supplementing renal graft preservation at subnormothermic temperatures with exogenous clinically approved H_2_S therapies, such as STS, may further enhance the mitochondrial and cytoprotective effects of subnormothermic renal graft preservation. These preservation techniques could eliminate the risk of cold IRI that is associated with the current standard of care, and, importantly, bridge the widening disparity between the supply and demand for transplantable kidneys.

## Data Availability

Data contained within the article.

## References

[1] Thurlow J.S., Joshi M., Yan G., Norris K.C., Agodoa L.Y., Yuan C.M., Nee R. (2021). Global Epidemiology of End-Stage Kidney Disease and Disparities in Kidney Replacement Therapy. Am. J. Nephrol..

[2] Deng Y., Li N., Wu Y., Wang M., Yang S., Zheng Y., Deng X., Xiang D., Zhu Y., Xu P. (2021). Global, Regional, and National Burden of Diabetes-Related Chronic Kidney Disease From 1990 to 2019. Front. Endocrinol..

[3] Lacroix J.D., Mahoney J.E., Knoll G.A. (2004). Renal transplantation using non-heart-beating donors: A potential solution to the organ donor shortage in Canada. Can. J. Surg..

[4] Himmelfarb J., Vanholder R., Mehrota R., Tonelli M. (2020). The current and future landscape of dialysis. Nat. Rev. Nephrol..

[5] Hariharan S., Israni A.K., Danovitch G. (2021). Long-Term Survival after Kidney Transplantation. N. Engl. J. Med..

[6] Steichen C., Giraud S., Bon D., Barrou B., Badet L., Salamé E., Kerforne T., Allain G., Roumy J., Jayle C. (2018). Barriers and advances in kidney preservation. BioMed Res. Int..

[7] van Eck van der Sluijs A., Vonk S., van Jaarsveld B.C., Bonenkamp A.A., Abrahams A.C. (2021). Good practices for dialysis education, treatment, and eHealth: A scoping review. PLoS ONE.

[8] Tonelli M., Wiebe N., Knoll G., Bello A., Browne S., Jadhav D., Klarenbach S., Gill J. (2011). Systematic review: Kidney transplantation compared with dialysis in clinically relevant outcomes. Am. J. Transplant..

[9] Akoh J.A. (2012). Kidney donation after cardiac death. World J. Nephrol..

[10] Resch T., Cardini B., Oberhuber R., Weissenbacher A., Dumfarth J., Krapf C., Boesmueller C., Oefner D., Grimm M., Schneeberger S. (2020). Transplanting Marginal Organs in the Era of Modern Machine Perfusion and Advanced Organ Monitoring. Front. Immunol..

[11] Min Y., Cheng L., Tu C., Li H., He D., Huang D., Chen D., Huang X., Chen F., Xiong F. (2021). Clinical characteristics of deceased hemodialysis patients affected by COVID-19. Int. Urol. Nephrol..

[12] Squires J.E., Grimshaw J.M., Taljaard M., Linklater S., Chassé M., Shemie S.D., Knoll G.A. (2014). Design, implementation, and evaluation of a knowledge translation intervention to increase organ donation after cardiocirculatory death in Canada: A study protocol. Implement. Sci..

[13] Hernandez-Alejandro R., Caumartin Y., Marotta P.J., Ghent C., Levstik M.A., Quan D., Muirhead N., House A.A., McAlister V., Jevnikar A.M. (2010). Kidney and liver transplants from donors after cardiac death: Initial experience at the London Health Sciences Centre. Can. J. Surg..

[14] Canadian Institute for Health Information (2021). CORR Annual Statistics. Organ Replacement in Canada. https://www.cihi.ca/en/organ-replacement-in-canada-corr-annual-statistics.

[15] Health Resources & Services Administration (2022). Learn About Donation. Organ Donation Statistics. https://www.organdonor.gov/learn/organ-donation-statistics.

[16] de Vries E.E., Snoeijs M.G., van Heurn E. (2010). Kidney donation from children after cardiac death. Crit Care Med..

[17] Salvadori M., Rosso G., Bertoni E. (2015). Update on ischemia-reperfusion injury in kidney transplantation: Pathogenesis and treatment. World J. Transplant..

[18] Batal I., De Serres S.A., Safa K., Bijol V., Ueno T., Onozato M.L., Iafrate A.J., Herter J.M., Lichtman A.H., Mayadas T.N. (2015). Dendritic cells in kidney transplant biopsy samples are associated with T cell infiltration and poor allograft survival. J. Am. Soc. Nephrol..

[19] Kosieradzki M., Rowiński W. (2008). Ischemia/Reperfusion Injury in Kidney Transplantation: Mechanisms and Prevention. Transplant. Proc..

[20] Salahudeen A.K., Huang H., Joshi M., Moore N.A., Jenkins J.K. (2003). Involvement of the mitochondrial pathway in cold storage and rewarming-associated apoptosis in human renal proximal tubular cells. Am J. Transplant..

[21] Green D.R., Kroemer G. (2004). The pathophysiology of mitochondrial cell death. Science.

[22] Rychkov D., Sur S., Sirota M. (2021). Molecular diversity of clinically stable human kidney allografts. JAMA Network Open.

[23] Nieuwenhuijs-Moeke G.J., Pischke S.E., Berger S.P., Sanders J.S.F., Pol R.A., Struys M.M.R.F., Ploeg R.J., Leuvenink H.G.D. (2020). Ischemia and reperfusion injury in kidney transplantation: Relevant mechanisms in injury and repair. J. Clin. Med..

[24] Elmi A., Mishabi M.A., Teymoori E., Nikbakht D., Sarraf N., Jabinian F., Khalilpour A., Askarkhah A., Rahmani V. (2021). A systematic review of the relationship between acute tubular necrosis and kidney transplantation. J. Ren. Inj. Prev..

[25] Hartzell S., Bin S., Cantarelli C., Haverly M., Manrique J., Angeletti A., Manna G.L., Murphy B., Zhang W., Levitsky J. (2020). Kidney Failure Associates with T Cell Exhaustion and Imbalanced Follicular Helper T Cells. Front. Immunol..

[26] Nita M., Grzybowski A. (2016). The Role of the Reactive Oxygen Species and Oxidative Stress in the Pathomechanism of the Age-Related Ocular Diseases and Other Pathologies of the Anterior and Posterior Eye Segments in Adults. Oxid. Med. Cell. Longev..

[27] Patel N.S., Chatterjee P.K., Di Paola R., Mazzon E., Britti D., De Sarro A., Cuzzocrea S., Thiemermann C. (2005). Endogenous interleukin-6 enhances the renal injury, dysfunction, and inflammation caused by ischemia/reperfusion. J. Pharmacol. Exp. Ther..

[28] Burne M.J., Elghandour A., Haq M., Saba S.R., Norman J., Condon T., Bennett F., Rabb H. (2001). IL-1 and TNF independent pathways mediate ICAM-1/VCAM-1 up-regulation in ischemia reperfusion injury. J. Leukoc. Biol..

[29] Meldrum K.K., Meldrum D.R., Meng X., Ao L., Harken A.H. (2002). TNF-alpha-dependent bilateral renal injury is induced by unilateral renal ischemia-reperfusion. Am. J. Physiol. Heart. Circ. Physiol..

[30] Mannon R.B. (2018). Delayed graft function: The AKI of kidney transplantation. Nephron.

[31] Wang W., Xie D., Hu X., Yin H., Liu H., Zhang X. (2017). Effect of Hypothermic Machine Perfusion on the Preservation of Kidneys Donated After Cardiac Death: A Single-Center, Randomized, Controlled Trial. Artif. Organs.

[32] Tingle S.J., Figueiredo R.S., Moir J.A., Goodfellow M., Talbot D., Wilson C.H. (2019). Machine perfusion preservation versus static cold storage for deceased donor kidney transplantation. Cochrane Database Syst. Rev..

[33] Chevalier R.L. (2016). The proximal tubule is the primary target of injury and progression of kidney disease: Role of the glomerulotubular junction. Am. J. Physiol. Renal Physiol..

[34] Dragun D., Hoff U., Park J.K., Qun Y., Schneider W., Luft F.C., Haller H. (2001). Prolonged cold preservation augments vascular injury independent of renal transplant immunogenicity and function. Kidney Int..

[35] Tozzi M., Franchin M., Soldini G., Ietto G., Chiappa C., Maritan E., Villa F., Carcano G., Dionigi R. (2013). Impact of static cold storage VS hypothermic machine preservation on ischemic kidney graft: Inflammatory cytokines and adhesion molecules as markers of ischemia/reperfusion tissue damage. Our preliminary results. Int. J. Surg..

[36] Hosgood S.A., Thompson E., Moore T., Wilson C.H., Nicholson M.L. (2018). Normothermic machine perfusion for the assessment and transplantation of declined human kidneys from donation after circulatory death donors. Br. J. Surg..

[37] Minor T., von Horn C., Gallinat A., Kaths M., Kribben A., Treckmann J., Paul A. (2020). First-in-man controlled rewarming and normothermic perfusion with cell-free solution of a kidney prior to transplantation. Am. J. Transplant..

[38] Burlage L.C., Tessier S.N., Etra J.W., Uygun K., Brandacher G. (2018). Advances in machine perfusion, organ preservation, and cryobiology: Potential impact on vascularized composite allotransplantation. Curr. Opin. Organ Transplant..

[39] van Smaalen T.C., Hoogland E.R., van Heurn L.W. (2013). Machine perfusion viability testing. Curr. Opin. Organ Transplant..

[40] Jochmans I., Moers C., Smits J.M., Leuvenink H.G., Treckmann J., Paul A., Rahmel A., Squifflet J.P., van Heurn E., Monbaliu D. (2010). Machine perfusion versus cold storage for the preservation of kidneys donated after cardiac death: A multicenter, randomized, controlled trial. Ann. Surg..

[41] Hoyer D.P., Gallinat A., Swoboda S., Wohlschläger J., Rauen U., Paul A., Minor T. (2014). Subnormothermic machine perfusion for preservation of porcine kidneys in a donation after circulatory death model. Transpl. Int..

[42] Bhattacharjee R.N., Patel S.V.B., Sun Q., Jiang L., Richard-Mohamed M., Ruthirakanthan A., Aquil S., Al-Ogaili R., Juriasingani S., Sener A. (2020). Renal protection against ischemia reperfusion injury: Hemoglobin-based oxygen carrier-201 versus blood as an oxygen carrier in ex vivo subnormothermic machine perfusion. Transplantation.

[43] Juriasingani S., Ruthirakanthan A., Richard-Mohamed M., Akbari M., Aquil S., Patel S., Al-Ogaili R., Whiteman M., Luke P., Sener A. (2021). Subnormothermic perfusion with h2s donor ap39 improves dcd porcine renal graft outcomes in an ex vivo model of kidney preservation and reperfusion. Biomolecules.

[44] Michel S.G., La Muraglia G.M., Madariaga M.L.L., Titus J.S., Selig M.K., Farkash E.A., Allan J.S., Anderson L.M., Madsen J.C. (2015). Twelve-hour hypothermic machine perfusion for donor heart preservation leads to improved ultrastructural characteristics compared to conventional cold storage. Ann. Transplant..

[45] Sage A.T., Richard-Greenblatt M., Zhong K., Bai X.H., Snow M.B., Babits M., Ali A., Baciu C., Yeung J.C., Liu M. (2021). Prediction of donor related lung injury in clinical lung transplantation using a validated ex vivo lung perfusion inflammation score. J. Heart Lung Transplant..

[46] Ali A., Wang A., Ribeiro R.V.P., Beroncal E.L., Baciu C., Galasso M., Gomes B., Mariscal A., Hough O., Brambate E. (2021). Static lung storage at 10 °C maintains mitochondrial health and preserves donor organ function. Sci. Transl. Med..

[47] Gwangwa M.V., Joubert A.M., Visagie M.H. (2019). Effects of glutamine deprivation on oxidative stress and cell survival in breast cell lines. Biol. Res..

[48] Yoo H.C., Yu Y.C., Sung Y., Han J.M. (2020). Glutamine reliance in cell metabolism. Exp. Mol. Med..

[49] Li T., Zhang Z., Kolwicz Jr S.C., Abell L., Roe N.D., Kim M., Tian R. (2017). Defective branched-chain amino acid catabolism disrupts glucose metabolism and sensitizes the heart to ischemia-reperfusion injury. Cell Metab..

[50] Silva M.A., Richards D.A., Bramhall S.R., Adams D.H., Mirza D.F., Murphy N. (2005). A study of the metabolites of ischemia-reperfusion injury and selected amino acids in the liver using microdialysis during transplantation. Transplantation.

[51] Erkasap S., Ates E. (2000). L-Arginine-enriched preservation solution decreases ischemia/reperfusion injury in canine kidneys after long-term cold storage. Nephrol. Dial. Transplant..

[52] Kwon Y.S., Foley J.D., Murphy C.J., McAnulty J.F. (2007). The effect of trophic factor supplementation on cold ischemia-induced early apoptotic changes. Transplantation.

[53] Ćelić T., Omrčen H., Španjol J., Bobinac D. (2018). Mechanisms of Bone Morphogenetic Protein-7 Protective Effects Against Cold Ischemia-Induced Renal Injury in Rats. Transplant. Proc..

[54] Ćelić T., Spanjol J., Grskovic A., Markic D., Prebilic I., Fuckar Z., Bobinac D. (2011). Bone morphogenetic protein-7 reduces cold ischemic injury in rat kidney. Transplant. Proc..

[55] Yang B., Hosgood S.A., Nicholson M.L. (2011). Naked small interfering RNA of caspase-3 in preservation solution and autologous blood perfusate protects isolated ischemic porcine kidneys. Transplantation.

[56] Moser M.A., Arcand S., Lin H.B., Wojnarowicz C., Sawicka J., Banerjee T., Luo Y., Beck G.R., Luke P.P., Sawicki G. (2016). Protection of the Transplant Kidney from Preservation Injury by Inhibition of Matrix Metalloproteinases. PLoS ONE.

[57] Sawicki G., Leon H., Sawicka J., Sariahmetoglu M., Schulze C.J., Scott P.G., Szczesna-Cordary D., Schulz R. (2005). Degradation of myosin light chain in isolated rat hearts subjected to ischemia-reperfusion injury: A new intracellular target for matrix metalloproteinase-2. Circulation.

[58] Wang W., Schulze C.J., Suarez-Pinzon W.L., Dyck J.R., Sawicki G., Schulz R. (2002). Intracellular action of matrix metalloproteinase-2 accounts for acute myocardial ischemia and reperfusion injury. Circulation.

[59] Ashenden M.J., Schumacher Y.O., Sharpe K., Varlet-Marie E., Audran M. (2007). Effects of Hemopure on maximal oxygen uptake and endurance performance in healthy humans. Int. J. Sports Med..

[60] Aburawi M.M., Fontan F.M., Karimian N., Eymard C., Cronin S., Pendexter C., Nagpal S., Banik P., Ozer S., Mahboub P. (2019). Synthetic hemoglobin-based oxygen carriers are an acceptable alternative for packed red blood cells in normothermic kidney perfusion. Am. J. Transplant..

[61] Mahboub P., Aburawi M., Karimian N., Lin F., Karabacak M., Fontan F., Tessier S.N., Markmann J., Yeh H., Uygun K. (2020). The efficacy of HBOC-201 in ex situ gradual rewarming kidney perfusion in a rat model. Artif. Organs.

[62] Juriasingani S., Jackson A., Zhang M.Y., Ruthirakanthan A., Dugbartey G.J., Sogutdelen E., Levine M., Mandurah M., Whiteman M., Luke P. (2021). Evaluating the Effects of Subnormothermic Perfusion with AP39 in a Novel Blood-Free Model of Ex Vivo Kidney Preservation and Reperfusion. Int. J. Mol. Sci..

[63] Mustafa A.K., Gadalla M.M., Snyder S.H. (2009). Signaling by gasotransmitters. Sci. Signal..

[64] Wang R. (2004). Signal Transduction and the Gasotransmitters: NO, CO, and H_2_S in Biology and Medicine.

[65] Mir J.M., Maurya R.C. (2018). A gentle introduction to gasotransmitters with special reference to nitric oxide: Biological and chemical implications. Rev. Inorg. Chem..

[66] Sener A., Tran K.C., Deng J.P., Garcia B., Lan Z., Liu W., Sun T., Arp J., Salna M., Acott P. (2013). Carbon monoxide releasing molecules inhibit cell death resulting from renal transplantation related stress. J. Urol..

[67] Dugbartey G.J., Alornyo K.K., Luke P.W., Sener A. (2021). Application of carbon monoxide in kidney and heart transplantation: A novel pharmacological strategy for broader use of suboptimal renal and cardiac grafts. Pharmacol. Res..

[68] Untereiner A.A., Wu L., Wang R. (2012). The role of carbon monoxide as a gasotransmitter in cardiovascular and metabolic regulation. Gasotransmitters: Physiology and Pathophysiology.

[69] Hosgood S.A., Bagul A., Kaushik M., Rimoldi J., Gadepalli R.S., Nicholson M.L. (2008). Application of nitric oxide and carbon monoxide in a model of renal preservation. British J. Surg..

[70] Beauchamp R.O., Bus J.S., Popp J.A., Boreiko C.J., Andjelkovich D.A., Leber P. (1984). A critical review of the literature on hydrogen sulfide toxicity. Crit. Rev. Toxicol..

[71] Hosgood S.A., Nicholson M.L. (2010). Hydrogen sulphide ameliorates ischaemia-reperfusion injury in an experimental model of non-heart-beating donor kidney transplantation. Br. J. Surg..

[72] Kimura H. (2015). Signaling molecules: Hydrogen sulfide and polysulfide. Antioxid. Redox. Signal..

[73] Zhang M.Y., Dugbartey G.J., Juriasingani S., Sener A. (2021). Hydrogen sulfide metabolite, sodium thiosulfate: Clinical applications and underlying molecular mechanisms. Int. J. Mol. Sci..

[74] Lobb I., Mok A., Lan Z., Liu W., Garcia B., Sener A. (2012). Supplemental hydrogen sulfide protects transplant kidney function and prolongs recipient survival after prolonged cold ischemia-reperfusion injury by mitigating renal apoptosis and inflammation. BJU Int..

[75] Lobb I., Davidson M., Carter D., Liu W., Haig A., Gunaratnam L., Sener A. (2015). Hydrogen sulfide treatment mitigates renal allograft ischemia-reperfusion injury during cold storage and improves early transplant kidney function and survival following allogeneic renal transplantation. J. Urol..

[76] Lobb I., Jiang J., Lian D., Liu W., Haig A., Saha M.N., Torregrossa N., Wood M.E., Whiteman M., Sener A. (2017). Hydrogen Sulfide Protects Renal Grafts Against Prolonged Cold Ischemia-Reperfusion Injury via Specific Mitochondrial Actions. Am. J. Transplant..

[77] Lobb I., Zhu J., Liu W., Haig A., Lan Z., Sener A. (2014). Hydrogen sulfide treatment ameliorates long-term renal dysfunction resulting from prolonged warm renal ischemia-reperfusion injury. Can. Urol. Assoc. J..

[78] Juriasingani S., Akbari M., Chan J.Y., Whiteman M., Sener A. (2018). H2S supplementation: A novel method for successful organ preservation at subnormothermic temperatures. Nitric Oxide.

[79] Dugbartey G.J., Juriasingani S., Zhang M.Y., Sener A. (2021). H2S donor molecules against cold ischemia-reperfusion injury in preclinical models of solid organ transplantation. Pharmacol. Res..

[80] Hendry-Hofer T.B., Ng P.C., Witeof A.E., Mahon S.B., Brenner M., Boss G.R., Bebarta V.S. (2019). A Review on Ingested Cyanide: Risks, Clinical Presentation, Diagnostics, and Treatment Challenges. J. Med. Toxicol..

[81] Yu Z., Gu L., Pang H., Fang Y., Yan H., Fang W. (2015). Sodium thiosulfate: An emerging treatment for calciphylaxis in dialysis patients. Case Rep. Nephrol. Dial..

[82] Farese S., Stauffer E., Kalicki R., Hildebrandt T., Frey B.M., Frey F.J., Uehlinger D.E., Pasch A. (2011). Sodium thiosulfate pharmacokinetics in hemodialysis patients and healthy volunteers. Clin. J. Am. Soc. Nephrol..

[83] Laplace N., Kepenekian V., Friggeri A., Vassal O., Ranchon F., Rioufol C., Gertych W., Villeneuve L., Glehen O., Bakrin N. (2020). Sodium thiosulfate protects from renal impairment following hyperthermic intraperitoneal chemotherapy (HIPEC) with Cisplatin. Int. J. Hyperthermia..

[84] Zhang M.Y., Dugbartey G.J., Juriasingani S., Akbari M., Liu W., Haig A., McLeod P., Arp J., Sener A. (2022). Sodium thiosulfate-supplemented UW solution protects renal grafts against prolonged cold ischemia-reperfusion injury in a murine model of syngeneic kidney transplantation. Biomed. Pharmacother..

